# Severe Bicytopenia and Neutropenic Fever in a Patient With COVID-19 Pneumonia: A Diagnostic Challenge

**DOI:** 10.7759/cureus.105583

**Published:** 2026-03-21

**Authors:** Tumodir Abdallah, Annmarie T Sajeev, Madapulli Wickrama, Kamal Khalil

**Affiliations:** 1 Department of Internal Medicine, Detroit Medical Center (DMC) Sinai-Grace Hospital, Detroit, USA

**Keywords:** covid-19, hematologic abnormalities, infectious disease, neutropenia, thrombocytopenia

## Abstract

Coronavirus disease 2019 (COVID-19) is a multisystemic viral infection that primarily affects the respiratory system but is widely recognized for its diverse extrapulmonary manifestations. Hematologic abnormalities, especially those associated with inflammation and disrupted hemostasis, are prevalent and have been extensively reported. However, few cases of profound neutropenia and severe thrombocytopenia have been documented in the literature, making their occurrence extremely rare. Herein, we present a rare case of a 60-year-old woman who presented with a two-week history of painful oral ulcers, fever, chills, sore throat, dyspnea, dry cough, palpitations, and anorexia. She was diagnosed with COVID-19 pneumonia and complicated by acute hypoxic respiratory failure, severe thrombocytopenia, and neutropenic fever. She was treated with supplemental oxygen, dexamethasone, broad-spectrum antibiotics, and granulocyte colony-stimulating factor (G-CSF), resulting in clinical and hematologic recovery.

## Introduction

Coronavirus disease 2019 (COVID-19), since its emergence, has been a virus that has posed significant diagnostic and therapeutic challenges due to its variety of clinical manifestations. Often presenting with respiratory failure, its associated symptoms can worsen the initial presentation or lead to new manifestations of the disease. Hematologic abnormalities, particularly lymphopenia and thrombocytopenia, are well described in association with COVID-19; however, neutropenia and severe thrombocytopenia are less commonly reported and reflect an aggressive disease course with poor prognostic implications. Progressive declines in blood cell counts may indicate an impending cytokine storm, severe systemic inflammatory response, immune-mediated destruction, or bone marrow myelosuppression secondary to uncontrolled immune activation [1,2]. We describe a case of COVID-19 complicated by neutropenia and profound thrombocytopenia, with a platelet count of approximately 16,000/µL, and the associated clinical manifestations. Understanding the systemic nature of the COVID-19 virus, specifically to the hematologic system, can lead to more consideration of broader diagnostic and management approaches in affected patients.

## Case presentation

A 60-year-old African American woman presented with a two-week history of progressive, painful oral ulcers involving the buccal mucosa and sublingual region, accompanied by fever, chills, sore throat, dyspnea, dry cough, palpitations, and anorexia. She denied additional symptoms and reported no sexual activity, recent medication changes, or toxic exposures. Her medical history was notable for hypertension, type 2 diabetes mellitus, hypothyroidism, psoriasis, chronic obstructive pulmonary disease (COPD) (not requiring home oxygen), and lumbar spinal stenosis. Surgical history included multiple joint replacements. Home medications were losartan, levothyroxine, liraglutide, metformin, albuterol, fluticasone, methotrexate 2.5 mg once a week, and folic acid supplementation. She had quit smoking one month prior to presentation and denied alcohol or illicit drug use. The patient denied any recent changes to her methotrexate regimen and reported that she had been taking the same dose for several years. She denied any prior history of COVID-19 infection and reported that she had not received a vaccination against COVID-19.

Initial vital signs include blood pressure of 119/72 mmHg, heart rate of 107 beats per minute, temperature of 37.8°C, respiratory rate of 20 breaths per minute, and oxygen saturation of 99% on room air. Physical examination revealed multiple aphthous ulcers along the inner perioral mucosa, bilateral buccal mucosa, and sublingual surface, associated with hard palate erythema and diffuse oral mucosal dryness. Lung examination revealed normal breath sounds without accessory muscle use or respiratory distress. No lymphadenopathy, hepatosplenomegaly, petechiae, or ecchymoses were observed. The remainder of the physical examination was unremarkable.

Initial laboratory evaluation was significant for a positive nasopharyngeal COVID-19 polymerase chain reaction (PCR) test, leukopenia (white blood cell {WBC} of 1.7 × 10⁹/L), absolute neutrophil count (ANC) of 0.7 × 10⁹/L, thrombocytopenia (platelet count of 36 × 10⁹/L), and hemoglobin of 12.9 g/dL with microcytosis (mean corpuscular volume {MCV} of 76.4 fL). Inflammatory markers were elevated, including C-reactive protein (CRP) of 156.8 mg/L and erythrocyte sedimentation rate (ESR) of 50 mm/hour. Additional findings included troponin of 20 ng/L, D-dimer of 1.54 µg/mL, ferritin of 466.4 ng/mL, lactate dehydrogenase (LDH) of 325 U/L, and haptoglobin of 484 mg/dL (laboratory results shown in Table 1). Electrocardiography demonstrated sinus tachycardia without ischemic changes.

**Table 1:** Laboratory tests done on admission and day 4 and day 8 of admission. PCR: polymerase chain reaction

Category	Test	Day 1	Day 4	Day 8	Units	Reference range
Hematologic	White blood cell count	1.7	0.9	27.9	×10⁹/L	3.5-10.6
	Absolute neutrophil count	0.7	0.2	19	×10⁹/L	1.58-7.13
	Platelet count	36	16	138	×10⁹/L	150-450
	Hemoglobin	12.9	12.4	12.3	g/dL	11.5-15.1
	Mean corpuscular volume	76.4	-	-	fL	82-97
	Reticulocyte percentage	0.6	-	-	%	0.5-2.0
	Absolute reticulocyte count	26,200	-	-	cells/µL	25,000-100,000
	Atypical lymphocyte percentage	0.2	-	-	%	0.0-0.29
Inflammatory/infectious markers	C-reactive protein	156.8	-	-	mg/L	<10
	Erythrocyte sedimentation rate	50	-	-	mm/hour	0-20
	Ferritin	466.4	-	-	ng/mL	11-306.8
	Lactate dehydrogenase	325	-	-	U/L	140-271
	Haptoglobin	484	-	-	mg/dL	44-215
Coagulation markers	D-dimer	1.54	-	-	µg/mL	<0.50
	Prothrombin time	11.5	-	-	Seconds	9.4-11.7
	Activated partial thromboplastin time	28.1	-	-	Seconds	23.1-33.1
	International normalized ratio	1.08	-	-	-	0.8-1.11
	Fibrinogen	623	-	-	mg/dL	167-467
	Fibrin monomers	Negative	-	-	-	Negative
Renal/metabolic	Creatinine	0.76	0.84	0.75	mg/dL	0.60-1.30
	Blood urea nitrogen	17	22	17	mg/dL	7-25
	Lactic acid	1.9	-	-	mmol/L	0.4-2.0
Hepatic function	Albumin	3.5	-	-	g/dL	3.5-5.7
	Alanine aminotransferase	17	-	-	U/L	7-52
	Aspartate aminotransferase	21	-	-	U/L	13-39
	Alkaline phosphatase	54	-	-	U/L	50-142
Cardiac marker	Troponin	20	-	-	ng/L	3-17
Endocrine/nutritional	Thyroid-stimulating hormone	1.96	-	-	mIU/L	0.45-5.33
	Vitamin B12	260	-	-	pg/mL	180-914
	Folic acid	21.1	-	-	ng/mL	>5.4
Iron studies	Serum iron	15	-	-	µg/dL	50-212
	Total iron-binding capacity	177	-	-	µg/dL	250-450
	Unsaturated iron-binding capacity	162	-	-	µg/dL	155-355
	Iron saturation	9	-	-	%	20-50
Trace element	Serum copper	134	-	-	µg/dL	80-158
Hematologic malignancy screening	Free kappa light chains	5.28	-	-	mg/L	0.33-1.94
	Free lambda light chains	3.10	-	-	mg/L	0.57-2.63
	Kappa/lambda ratio	1.7	-	-	-	0.26-1.65
Autoimmune testing	Antinuclear antibody	Not detected	-	-	-	Not detected
	Rheumatoid factor	18	-	-	IU/mL	<15
Infectious disease testing	Syphilis	<10	-	-	-	<0.90
	HIV antigen/antibody	Nonreactive	-	-	-	Nonreactive
	Hepatitis B surface antigen	Negative	-	-	-	Negative
	Hepatitis C antibody	Negative	-	-	-	Negative
	Parvovirus	Not detected	-	-	-	Not detected
	Cytomegalovirus PCR	Not detected	-	-	-	Not detected
	Influenza A/B PCR	Not detected	-	-	-	Not detected
Toxicology	Alcohol level	<10	-	-	mg/dL	<10

**Figure 1:**
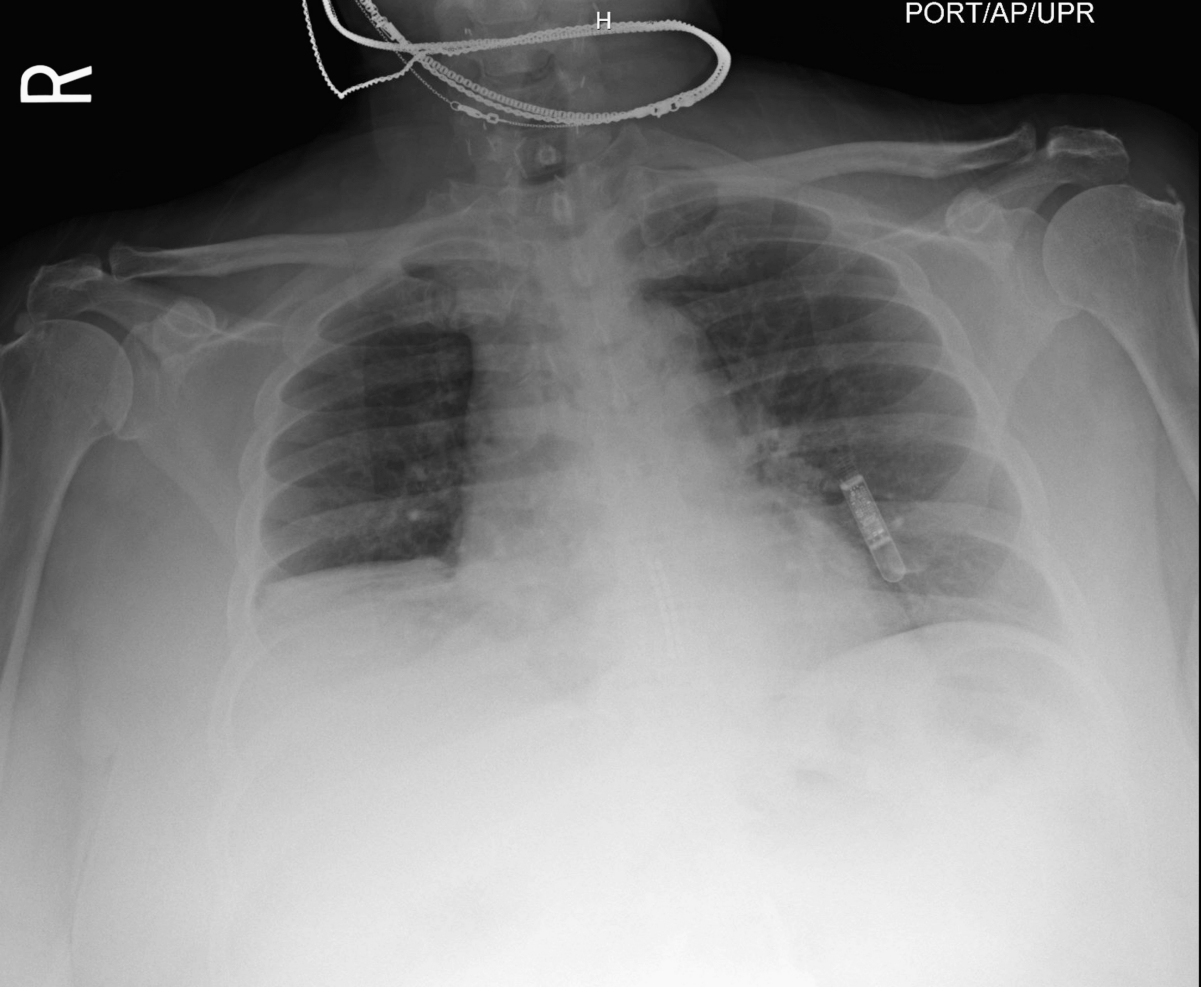
CXR on hospital day 1: no acute cardiopulmonary abnormalities. CXR: chest radiograph

**Figure 2:**
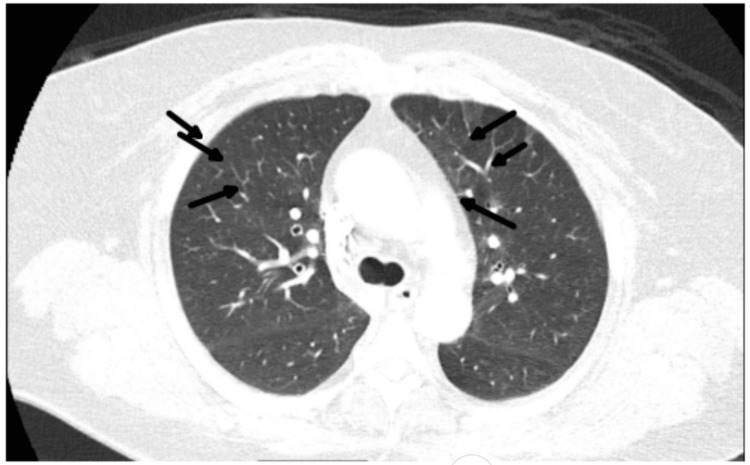
Chest CT angiography on hospital day 1: excluded pulmonary embolism but showed right middle lobe atelectasis and bilateral patchy parenchymal opacities suggestive of inflammatory changes. CT: computed tomography

Chest radiograph (CXR) revealed no acute cardiopulmonary abnormalities (Figure 1). Chest computed tomography (CT) angiography excluded pulmonary embolism but showed right middle lobe atelectasis and bilateral patchy parenchymal opacities suggestive of inflammatory changes (Figure 2).

**Figure 3:**
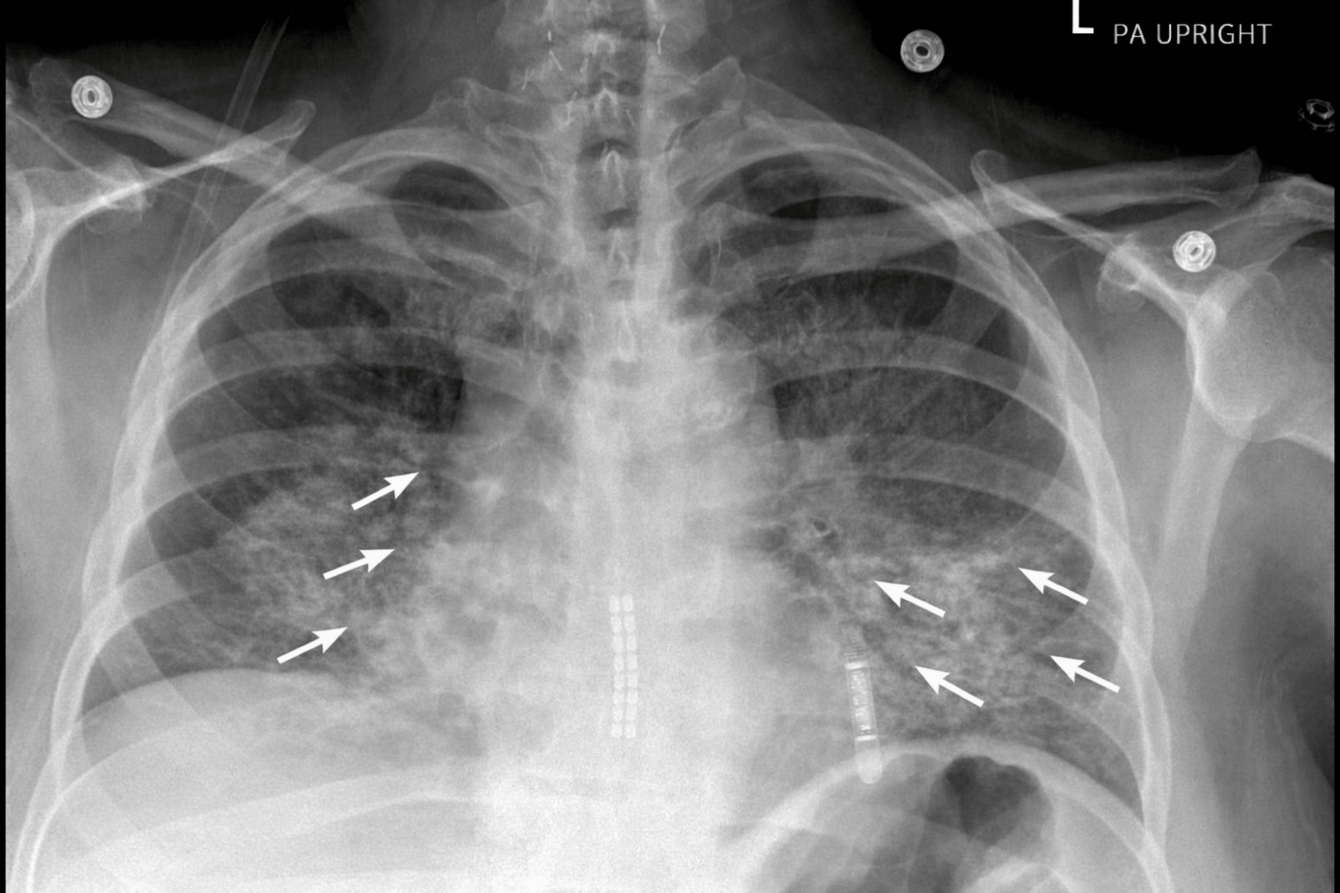
CXR on hospital day 4: new bilateral infiltrates with vascular congestion. CXR: chest radiograph

Supportive management was initiated, including intravenous fluids, bronchodilator therapy, and pain management. On hospital day 4, she developed hypoxia with oxygen saturation decreasing to 85% and recurrent fevers up to 39.4°C. Repeat laboratory testing revealed worsening bicytopenia with WBC of 0.9 × 10⁹/L, ANC of 0.2 × 10⁹/L, and platelet count of 16 × 10⁹/L, without evidence of active bleeding. Repeat CXR revealed new bilateral infiltrates with vascular congestion (Figure 3).

She was placed on 6 L/minute oxygen via nasal cannula (NC) and initiated on dexamethasone 6 mg daily for 10 days. Given the concern for neutropenic fever, broad-spectrum antimicrobial therapy with cefepime and vancomycin was administered for six days, and the patient received three doses of 400 μg of granulocyte colony-stimulating factor (G-CSF). She improved gradually with the resolution of fever and the progressive recovery of oxygenation to 4 L/minute via NC. Blood cultures were negative. By hospital day 8, hematologic parameters and the patient's symptoms had significantly improved, with WBC of 27.9 × 10⁹/L, ANC of 19 × 10⁹/L, and platelet count of 138 × 10⁹/L. Hematology/oncology was consulted and given the negative diagnostic workup and the patient's clinical deterioration from COVID-19 infection by hospital day 4; the hematologic abnormalities were considered most consistent with COVID-19-related changes rather than a primary hematologic disorder. She was discharged home on 4 L/minute supplemental oxygen with instructions to discontinue methotrexate and to follow outpatient instructions.

## Discussion

COVID-19 is a respiratory infection that has demonstrated a variety of patient presentations from 2019 to the present. Although the most common presentation has been an acute respiratory prodrome, recent reports have described hematologic abnormalities associated with COVID-19 infection. One rare hematologic abnormality is neutropenia. Neutropenia is defined as a decrease in neutrophils to approximately <1,500 cells/µL. Neutrophils play a critical role in the immune system, and their presence is essential for fighting infections and maintaining host defense. A decrease in neutrophil count can result in weakened immunity, increased susceptibility to infections, the worsening of baseline comorbidities, and prolonged recovery time. Neutropenia with concurrent thrombocytopenia following COVID-19 infection is an even rarer occurrence. Thrombocytopenia is defined as a platelet count of below 150,000/µL and can impair primary hemostasis, resulting in prolonged bleeding time. Although thrombocytopenia has been reported in association with COVID-19, the severity observed in our patient (~16,000/µL), which is classified as life-threatening, represents one of the more severe thrombocytopenic presentations not yet well described in the literature. The combined presence of neutropenia and thrombocytopenia during or following severe acute respiratory syndrome coronavirus 2 (SARS-CoV-2) infection may lead to significant complications and worsen the clinical course. The early recognition of the association between COVID-19 and these hematologic abnormalities may facilitate a more rapid diagnostic approach and timely treatment [1-3].

The mechanism by which COVID-19 may cause neutropenia and thrombocytopenia is not clearly defined; however, several mechanisms have been proposed. One such mechanism involves the reprogramming of neutrophils, as suggested in a study conducted at Johns Hopkins University [4]. The study proposed that SARS-CoV-2 may induce phenotypic changes in neutrophils, transforming them into polymorphonuclear myeloid-derived suppressor cells (PMN-MDSCs) [4]. These cells are known to suppress T-cell activity, thereby further weakening the immune response and potentially contributing to more severe manifestations of COVID-19. Most reported cases of COVID-19-associated neutropenia have demonstrated elevated inflammatory markers, including ESR, CRP, LDH, and ferritin, similar to those observed in our patient [3]. The prolonged inflammatory response triggered by the virus can result in autoimmunity and its associated immune activation of autoantibodies that suppress the bone marrow cells. This mechanism has been described in association with several other viral infections, including influenza, cytomegalovirus (CMV), and Epstein-Barr virus (EBV) [3]. Additionally, the direct infection of bone marrow progenitor cells during viremia may result in the maturation arrest of the myeloid cell line. Given these proposed mechanisms, our case further supports the need for continued investigation into the hematopoietic effects of SARS-CoV-2 infection and its potential to induce transient or severe bone marrow suppression [5].

The treatment of COVID-19-associated neutropenia has depended on the severity of patient presentation and the timeline of neutropenia. In most reported cases, neutropenia has been associated with mild-to-moderate respiratory presentations. The neutropenia was transient and resolved spontaneously after the resolution of the viral infection, although in some cases this resolution was delayed [6]. However, several case reports have described the use of G-CSF to treat neutropenia. The use of G-CSF in this context has been debated. In certain cases, G-CSF administration resulted in the rapid resolution of neutropenia. Conversely, other reports have demonstrated that neutrophil recovery following G-CSF treatment was associated with an excessive inflammatory response, particularly in individuals with malignancies or other severe comorbidities. G-CSF increases neutrophil counts and also promotes cytokine production. In patients who already have an elevated inflammatory burden secondary to COVID-19, this may precipitate more severe or symptomatic clinical manifestations following G-CSF treatment [7]. Notably, some research has targeted monoclonal antibodies against G-CSF, such as lenzilumab, as a therapeutic strategy in COVID-19 to mitigate the inflammatory response [7]. Currently, G-CSF may be considered selectively as one of the treatments for COVID-19-associated neutropenia; however, careful consideration should be given to the patient's baseline inflammatory state, particularly in the presence of preexisting comorbidities that may further amplify inflammation.

## Conclusions

COVID-19 has demonstrated a continually evolving spectrum of clinical presentations. Hematologic abnormalities, particularly neutropenia and severe thrombocytopenia, represent important laboratory findings that should prompt clinical concern. Given the wide variability in patient demographics and disease manifestations, routine evaluation for hematologic derangements should be considered in all individuals infected with SARS-CoV-2. The underlying mechanisms driving these abnormalities remain incompletely understood, underscoring the need for further investigation into the complex interactions between the virus and host immune response. The improved understanding of these processes, coupled with the early recognition of hematologic abnormalities, may facilitate timely intervention, optimize management, and potentially shorten recovery times.
